# Technical development and implementation of 3D‐QALAS on a 1.5T MR‐Linac for the brain: A prospective R‐IDEAL stage 0/1 technology development report

**DOI:** 10.1002/acm2.70886

**Published:** 2026-10-28

**Authors:** Lucas McCullum, Ashley Harrington, Natalie West, Grace Nehring, Ziyu Fu, Ergys Subashi, Brian A. Taylor, Ken‐Pin Hwang, Clifton D. Fuller

**Affiliations:** ^1^ UT MD Anderson Cancer Center UTHealth Houston Graduate School of Biomedical Sciences Houston Texas USA; ^2^ Department of Radiation Oncology The University of Texas MD Anderson Cancer Center Houston Texas USA; ^3^ Department of Imaging Physics The University of Texas MD Anderson Cancer Center Houston Texas USA; ^4^ Department of Radiation Physics The University of Texas MD Anderson Cancer Center Houston Texas USA

**Keywords:** 3D‐QALAS, adaptive radiation therapy, brain, cancer, MRI, MR‐Linac, QALAS, quantitative imaging, radiation therapy, radiotherapy, SyMRI, SyntheticMR

## Abstract

**Background:**

Quantitative relaxometry of the brain on the integrated MRI / linear accelerator (MR‐Linac) at high isotropic reconstructed resolution is currently limited due to prohibitively long scan times and limited field‐of‐views.

**Purpose:**

The purpose of this study was to assess the technical feasibility of the 3D‐QALAS technique on the 1.5T MR‐Linac which can acquire whole‐brain 1 mm isotropic quantitative T1, T2, and PD maps along with multiple synthetic images in a 7‐minute acquisition time.

**Methods:**

A 1 mm isotropic 3D‐QALAS acquisition was scanned in both phantoms and five healthy volunteers on the 1.5T Elekta Unity MR‐Linac device with scan times around seven minutes. A test‐retest protocol across five independent sessions for the phantom was conducted. The Lin's Concordance Correlation Coefficient (LCCC), repeatability, and reproducibility between measured and reference quantitative T1, T2, and PD values were determined in the phantom. Distortion was also studied. Vendor‐provided reconstruction through SyMRI was performed to extract synthetic images and brain volume metric assessments in the healthy volunteers.

**Results:**

The slope and LCCC between the measured and phantom reference values was 1.02 (1.00), 1.09 (0.90), and 0.99 (1.00) for T1, T2, and PD, respectively. Median distortion across the phantom remained below 2 mm. The repeatability and reproducibility coefficient‐of‐variation (CoV) was under 8% for all measured values. The measured brain volumes in the healthy volunteer was within expected age‐adjusted reference values.

**Conclusions:**

The technical feasibility of using 3D‐QALAS on the integrated 1.5T MR‐Linac was confirmed. Applying this technique to the adaptive radiation therapy workflow of the brain will provide new opportunities to integrate quantitative imaging relaxometry biomarkers at 1 mm isotropic reconstructed resolution.

## 1 INTRODUCTION

The combined magnetic resonance imaging (MRI) linear accelerator (Linac), or MR‐Linac, has seen increasing application in the treatment of cancers of the brain.^1^, ^2^, ^3^ On the MR‐Linac, the increased contrast resolution compared to computed tomography (CT) leads to improved contouring accuracy across the dice score, Hausdorff distance, and mean distance to agreement metrics.^4^ Even though contouring could be done on just the high‐resolution anatomic images, quantitative imaging can be supplemented to improve accuracy^5^, ^6^ and similar spatial resolutions should be used between the two images to reduce spatial blurring.^7^, ^8^ Further, acquiring at small voxel sizes will allow for stereotactic radiosurgery (SRS) treatments of the brain.^9^ More formerly, the consensus statement from the German Society for Radiation Oncology (DEGRO) and for physics and technology in stereotactic radiotherapy of the German Society for Medical Physics (DGMP) recommends a maximum geometric accuracy of 1 mm for SRS.^10^ Another motivation for high resolution quantitative imaging is for the identification of brain metastases which, in a recent consensus recommendation, should be imaged in at most 1.5 mm resolution on 1.5T and 1 mm resolution on 3T MRI scanners^11^ which was corroborated by another study from Cancer Care Ontario.^12^

One possible solution to high resolution multiparametric quantitative mapping on the MR‐Linac is the 2D multi‐dynamic multi‐echo (MDME) sequence^13^ from SyntheticMR^14^ (Linköping, Sweden), evolved from its original implementation,^15^ which can simultaneously quantify T1, T2, and PD along with synthetically‐generate contrast‐weighted images through the accompanying post‐processing software, SyMRI. In a previous study, McCullum et al. studied the technical feasibility of the 2D‐MDME sequence on the 1.5T MR‐Linac (Unity; Elekta AB; Stockholm, Sweden) and found mean bias in phantoms under 11% and correlations above 0.97 across multiple repeats.^16^ In a follow‐up study, McCullum et al. optimized the 2D‐MDME sequence across varying acquisition parameters and found that a minimum of a 3 mm slice thickness while preserving a 1 mm in‐plane resolution is recommended to maintain minimal noise as measured by the coefficient of variation in both phantoms and healthy volunteers.^17^ Although promising, a clear gap exists to reduce the slice thickness to the in‐plane spatial resolution of 1 mm, however this is not possible using the previously studied 2D‐MDME sequence on the MR‐Linac. Fortunately, a 3D version of the sequence capable of 1 mm isotropic acquisitions, namely Quantification using an interleaved Look‐Locker Acquisition Sequence with T2 preparation pulse (3D‐QALAS) / T2‐prepared Multiple Inversion Recovery (TPMID) / SyntAc on Philips systems, has been developed.^18^ This technique originally showed clinical acceptability in cardiac applications,^19^, ^20^ however newer translations have demonstrated its quantitative accuracy and repeatability in the brain in both multi‐center^21^ and multi‐vendor studies^22^ as well as in the breast^23^ and liver.^24^ Further, its acquisition time has been accelerated by integrating compressed sensing reconstruction leading to clinically feasible scan times^25^, ^26^, ^27^ while improving repeatability^28^ compared to traditional techniques like Driven Equilibrium Single Pulse Observation of T1 and T2 (DESPOT^29^). Another benefit of 3D‐QALAS compared to 2D‐MDME is its increased robustness to the magnetization transfer effect which reduces the quantitative T1 values by up to 35% in the 2D‐MDME sequence.^30^, ^31^

At the time of this writing, no studies to the author's knowledge have evaluated the technical feasibility of the 3D‐QALAS sequence on the MR‐Linac device. Therefore, in this study, our goal is to characterize the quantitative accuracy and repeatability of the 3D‐QALAS sequence on a 1.5T MR‐Linac for longitudinal biomarker study applications in the brain in addition to its geometric accuracy for precise targeting and segmentation pipelines in both phantoms and healthy volunteers. This will be presented using the radiotherapy‐predicate studies, idea, development, exploration, assessment, and long‐term study (R‐IDEAL) framework, as recommended by the MR‐Linac Consortium, completing Stage 0 (radiotherapy predicate studies) and Stage 1 (first time use) systematic evaluations.^32^

## 2 METHODS

### 2.1 MRI acquisition parameters

To evaluate the performance of the 3D‐QALAS sequence, MRI scans were performed on a 1.5T MR‐Linac (Unity; Elekta AB; Stockholm, Sweden) running Philips version R5.8.1. The 3D‐QALAS sequence is not available clinically on Philips version R5.8.1 at the time of this writing, but it is available on Philips version > R12.1.1, therefore the relevant pulse programming environment (PPE) code was backported to version R5.8.1 and compiled into a research patch. In addition, several clinical science keys (CSKs), primarily related to cardiac synchronization and external monitoring devices, had to be overridden in the research patch to run on our MR‐Linac system. The relevant acquisition parameters are shown in **Table** **1** which were slightly modified from the SyMRI version 15.0.19 recommendations for Philips scanners. This yielded dynamic times of 0, 130.5, 1026, 1922, and 2817 ms.

**TABLE 1:** Acquisition parameters across all scanners utilized in this study.

	1.5T Elekta Unity
Software Version	R5.8.1
Gradient Strength (mT/m)	15
Gradient Slew Rate (T/m/s)	65
Field‐of‐View (mm^3^)	256 × 256 × 200
Acquisition Matrix Size	128 × 128 × 200
Slice Gap (mm)	−1
Acquired Voxel Size (mm^3^)	2 × 2 × 2
Reconstruction Matrix Size	256 × 256 × 200
Reconstructed Voxel Size (mm^3^)	1 × 1 × 1
Uniformity Correction	CLEAR
Slice Oversampling	Default (1.4)
Slice Orientation	Transverse
Fold‐Over Direction	AP
Sequence	
Type	3D TFE
TFE Factor	113
Startup Echoes	0
Profile Order	Low‐High
Turbo Direction	Radial
TR / TE (ms)	6.5 / 4.6 (In‐Phase)
Flip Angle (°)	4
Bandwidth (Hz / Pixel)	1206 (Maximum)
Acceleration	
Type	CS‐SENSE
Factor	1.25
Denoising	Strong
TFE Prepulse	
Type	Invert
Delay (ms)	130.5
T2 Preparation Pulse	
Echo Time (ms)	100
Refocusing Pulses	4
Cardiac Synchronization	
Type	Internal Trigger
Cycle Duration (ms)	895.5
Fat Suppression	No
Water Suppression	No
NSA	1
Acquisition Time (m:ss)	7:30

Abbreviations: CLEAR, Constant LEvel AppeaRance; AP, anterior to posterior; CS‐SENSE, Compressed Sensing (CS) SENSitivity Encoding (SENSE); NSA, number of signal averages; TFE, turbo field echo; TR, repetition time; TE, echo time.

### 2.2 MRI acquisition methodology

Previously developed in collaboration with the National Institute of Standards and Technology (NIST) and the International Society of Magnetic Resonance in Medicine (ISMRM), the CaliberMRI “NIST/ISMRM” Premium System Phantom Model 130 phantom (CaliberMRI; Boulder, CO, USA) was used as a reference for NIST‐traceable T1, T2, and PD values and to assess geometric accuracy using the built‐in fiducial array.^33^, ^34^ The temperature was confirmed to be near 20°C in accordance with the phantom reference values prior to scanning using an analog thermometer placed next to the bore. Since reference data for this phantom was not available at 1.5T at the time of this writing, but only at 3T, we used a previous version of this phantom with both 1.5T and 3T reference data and created a rough conversion factor for quantitative T1, T2, and PD using a linear line‐of‐best‐fit. The resulting conversion factors were 0.93*x + 15.43 ms (*r^2^* = 0.9990) for quantitative T1, 1.06*x + 26.08 ms (*r^2^* = 0.9964) for quantitative T2, and 1*x + 0 pu (*r^2^* = 1) for quantitative PD. To evaluate reproducibility, the 3D‐QALAS sequence was acquired across five separate scanning sessions in the NIST/ISMRM phantom and in one session for five healthy volunteers. To evaluate repeatability in phantoms within each session, two repetitions were performed per session in a test‐retest method^35^ without breaks between repetitions. The phantom was removed from the table and repositioned between sessions which took approximately five minutes.

### 2.3 Data collection and image processing

The resulting magnitude images were processed using the SyntheticMR post‐processing software, SyMRI (StandAlone 0.61.0; SyntheticMR AB; Linköping, Sweden) developed for both the 2D‐MDME and 3D‐QALAS sequences. Since the research patch did not have access to the reconstruction code, it did not correctly assign the temporal position identifier (0020,0100), number of temporal positions (0020,0105), and T2prep time (2005,1712) DICOM tags, so these were modified following image acquisition in accordance with the SyMRI DICOM Conformance Statement using Python 3.12.10. The total SyMRI reconstruction time was ∼45 seconds (30s for DICOM file loading, 10s for curve fitting, and 5s for automatic cranial segmentations).

The geometric accuracy was evaluated using the NIST/ISMRM phantom by determining the coordinates of the center of each vial of the built‐in fiducial array and comparing those coordinates to their expected locations^34^ using the center coordinate of a circular region‐of‐interest (ROI) around the fiducial in 3D Slicer^36^ (https://www.slicer.org/). For the phantom analysis, a circular ROI was created in each vial across a single transverse slice of the reconstructed quantitative T1 map using 3D Slicer. A margin around the edge was left to account for ringing artifacts and partial volume effects.^34^ For the OOI analysis in the healthy volunteer, the white matter, grey matter, and whole brain was automatically contoured using SyMRI and the resulting mean and standard deviation of the quantitative T1, T2, and PD values inside each structure were extracted. SyMRI also produced the estimated white matter,^37^ grey matter,^37^ cerebral spinal fluid (CSF^37^), myelin,^38^ and non‐aqueous content^38^ maps which further yielded the calculated intracranial volume (ICV, white matter + grey matter + CSF + region growing algorithm to skull barrier assuming 50% PD), brain parenchymal volume (BPV, ICV—CSF), myelin‐correlated component (MyC^38^), myelin parenchymal fraction (MyCPF, MyC / BPV), brain parenchymal fraction (BPF, BPV / ICV). These measured values were compared to references curves derived from a combination of the results from McAllister et al. 2017 for children^39^ and Vågberg et al. 2016 for adults.^40^ Additional synthetic T1‐weighted, T2‐weighted, T2‐weighted fluid attenuated inversion recovery (FLAIR), proton density‐weighted, phase‐sensitive inversion recovery (PSIR), and double inversion recovery (DIR) with white matter suppression maps were generated for qualitative analysis.

### 2.4 Statistical analysis

All statistical methodology and analytic reporting were formulated using the Statistical Analyses and Methods in the Published Literature (SAMPL) guidelines.^41^ Distortion was assessed in each vial by determining the center location of each fiducial, calculating its distance from the center fiducial from the image, and comparing these measurements to the nominal distance of 40 mm between orthogonal fiducials. The mean and standard deviation of each vial inside the NIST/ISMRM phantom were extracted and compared to manufacturer reference values. The Lin's Concordance Correlation Coefficient^42^ (LCCC) was used to evaluate direct agreement to reference values while a Bland‐Altman plot^43^ was used to assess bias. In phantoms, the coefficient of variation (CoV) was used to assess repeatability, reproducibility, and noise within the uniform vials of the phantom. For all calculations, analysis was restricted to clinical ranges of T1 (200–2500 ms), T2 (35–120 ms), and PD (20–120 pu).

## 3 RESULTS

### 3.1 Phantom analysis

The measured geometric distortion using the built‐in fiducial array of the NIST/ISMRM is shown in Figure 1. The distortion at the center was 0 mm since this was used as the reference point. Since the fiducial points were separated by 40 mm, this was the next lowest distance from the center where the median [interquartile range (IQR)] was 1.83 [1.31 – 2.29] mm. The further the fiducials were from the center, the lower the metrics changed with the largest distortion seen at 89.44 mm from the center, the furthest measured point, with a median [IQR] of 1.98 [1.49–2.40] mm.

**FIGURE 1:**
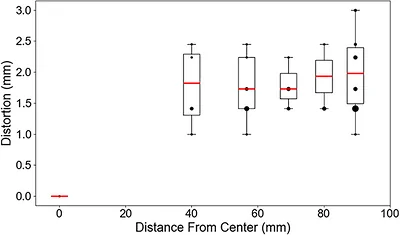
The quantified geometric distortion as a function of distance from the center fiducial point of the NIST/ISMRM phantom in boxplot form with overlayed scatter plot with the dot size representing the number of measurements at that coordinate.

After the geometric distortion was quantified and deemed clinically acceptable, the concordance between the measured quantitative T1, T2, and PD values against the NIST/ISMRM reference values was computed using all ten measurements (five sessions with one test‐retest per session) as shown in Figure 2. The linear slopes of agreement for the quantitative T1, T2, and PD were 1.02 (LCCC = 1.00), 1.09 (LCCC = 0.90), and 0.99 (LCCC = 1.00), respectively. Both the measured quantitative T1 and PD were strongly linear while the measured quantitative T2 had positive bias against the NIST/ISMRM phantom at both low and high T2 values at the edges of the chosen clinical range of 35–120 ms. If the quantitative T1 and PD plots were extended to lower values, then a stabilization of the measured values would be seen, indicating that the chosen minimum clinical range of 200 ms and 20 pu were lower limits for the 3D‐QALAS sequence at the current acquisition parameters.

**FIGURE 2:**
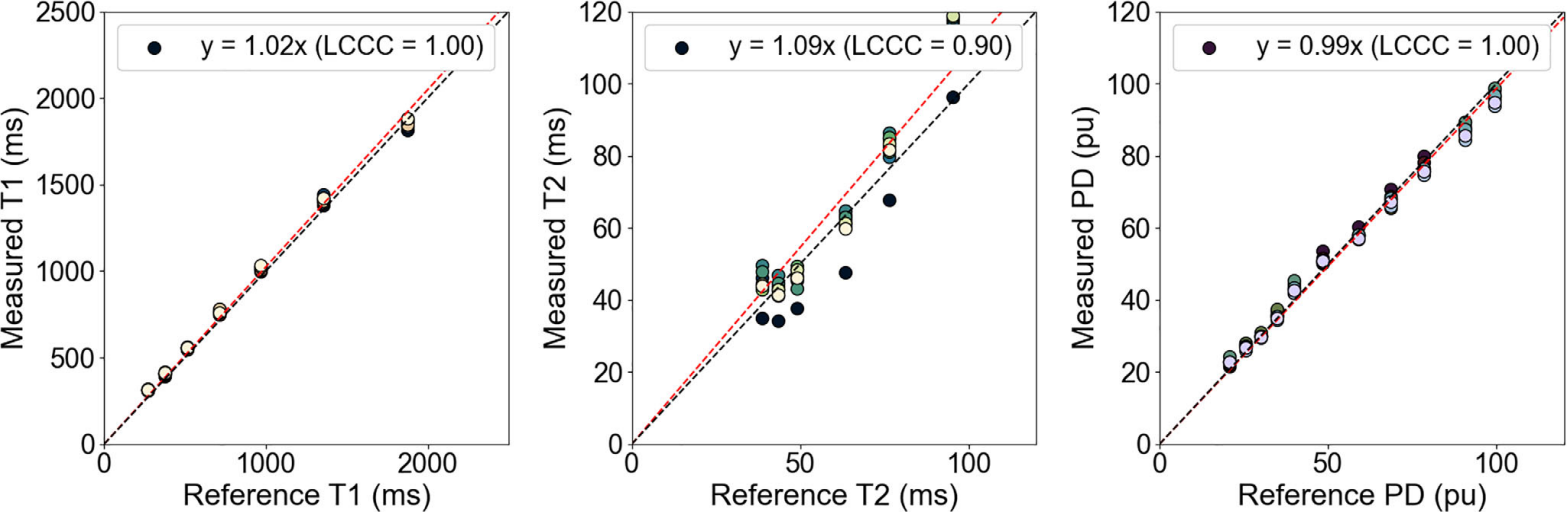
The correlation plot between the measured and reference quantitative T1 (left), T2 (center), and PD (right) across all ten measurements (five sessions with one test‐retest per session) of the 3D‐QALAS sequence on the 1.5T MR‐Linac. LCCC = Lin's Concordance Correlation Coefficient.

The corresponding Bland‐Altman plots for Figure 2 is shown in Figure 3. All 95% limits of agreement included the 0 value while a roughly 50 ms positive systematic bias in the measured quantitative T1 can be seen at T1 values less than 1500 ms. Unexpectedly, almost half of the measured quantitative T1 values near 2000 ms exceeded the 95% limits of agreement indicating that this may be the edge of confident quantitative assessment. A similar trend was observed for the quantitative T2 values above 100 ms with a slightly positive systematic bias. Almost all of the measured quantitative PD values remained within the 95% limits of agreement which was centered around 0 pu with a range of 5 pu in both the positive and negative bias directions with a potential for proportional bias. The corresponding ranges for the quantitative T1 and T2 were 50 and 20 ms in either direction, respectively.

**FIGURE 3:**
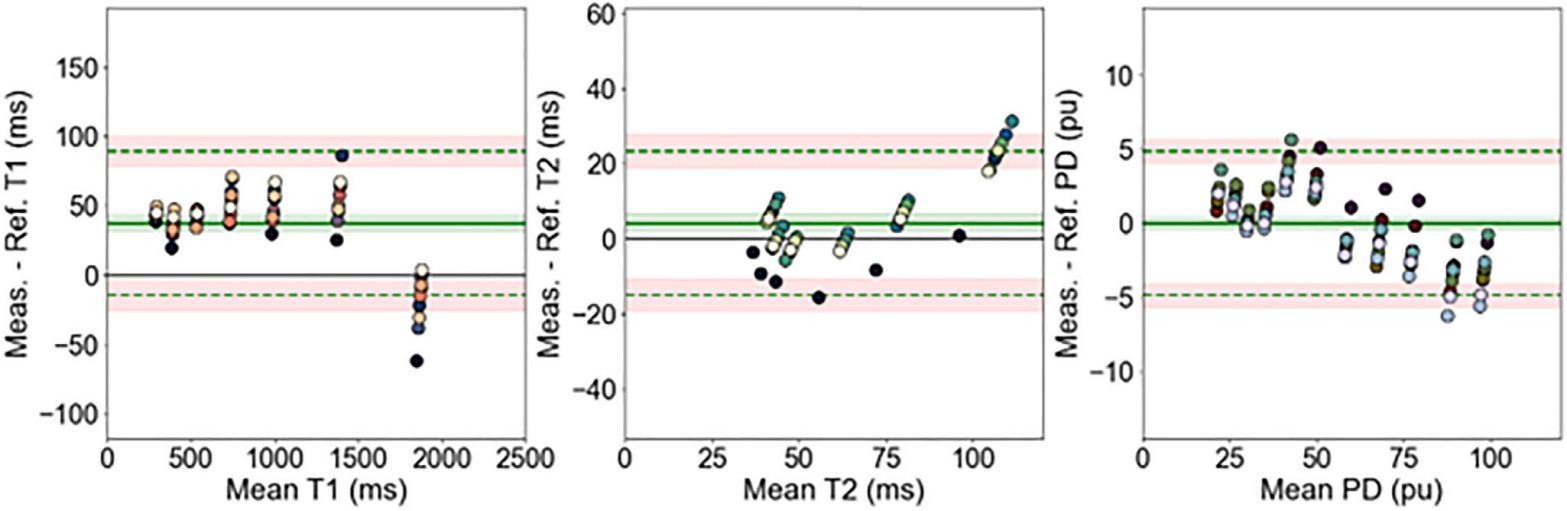
The Bland‐Altman plot between the measured and reference quantitative T1 (left), T2 (center), and PD (right) across all ten measurements (five sessions with one test‐retest per session) of the 3D‐QALAS sequence on the 1.5T MR‐Linac. The solid green line represents the mean across all biases while the dashed green lines represent the 95% limits of agreement. The shaded green and red regions represent the 95% confidence intervals on the sample mean and limits of agreement, respectively.

The corresponding repeatability of the two repetitions averaged across the five sessions and the reproducibility of the five sessions averaged across the two repetitions for the measured quantitative T1, T2, and PD is shown in Figure 4. Both the repeatability and reproducibility was under 2% for the quantitative T1 values while being under 3% for the quantitative PD values and under 8% for the quantitative T2 values. For all vials tested, the reproducibility remained higher than the repeatability. This difference was minimized for the quantitative T1 values where they were nearly identical and maximized for the quantitative PD values where the reproducibility was nearly twice as high. The quantitative T2 values fell within these two trends.

**FIGURE 4:**
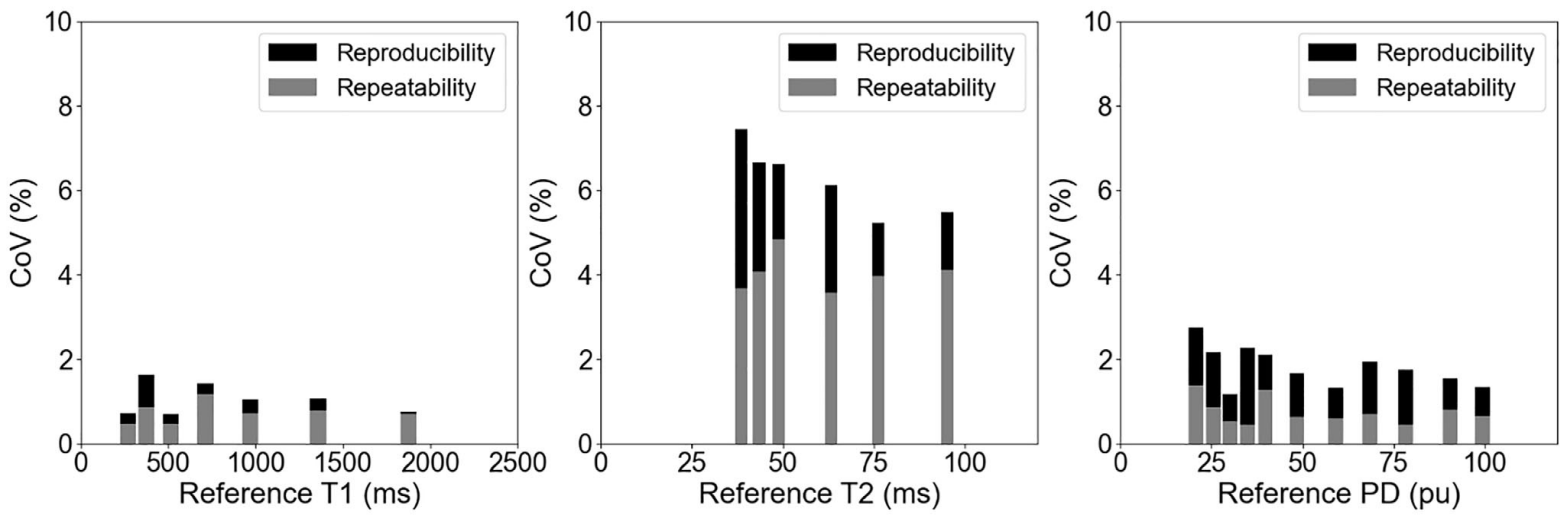
The repeatability of the two repetitions averaged across the five sessions and the reproducibility of the five sessions averaged across the two repetitions for the quantitative T1 (left), T2 (center), and PD (right) from the 3D‐QALAS sequence on the 1.5T MR‐Linac as measured by the CoV.

### 3.2 Healthy volunteer analysis

The quantitative T1, T2, and PD maps in axial, coronal, and sagittal orientations in the healthy volunteer are shown in Figure 5. The primary purpose of this figure was to demonstrate the benefits of the isotropic spatial resolution on different viewing orientations without the blurry appearance seen in 2D‐MDME which traditionally has thick (∼3–4 mm) slices. This healthy volunteer had a permanent retainer which led to the signal void in the oral cavity for all images shown for this volunteer. Slightly higher image noise can be seen in the quantitative T2 maps which was reflected in the previously shown concordance, repeatability, and reproducibility plots where is performed the worst compared to the quantitative T1 and PD.

**FIGURE 5:**
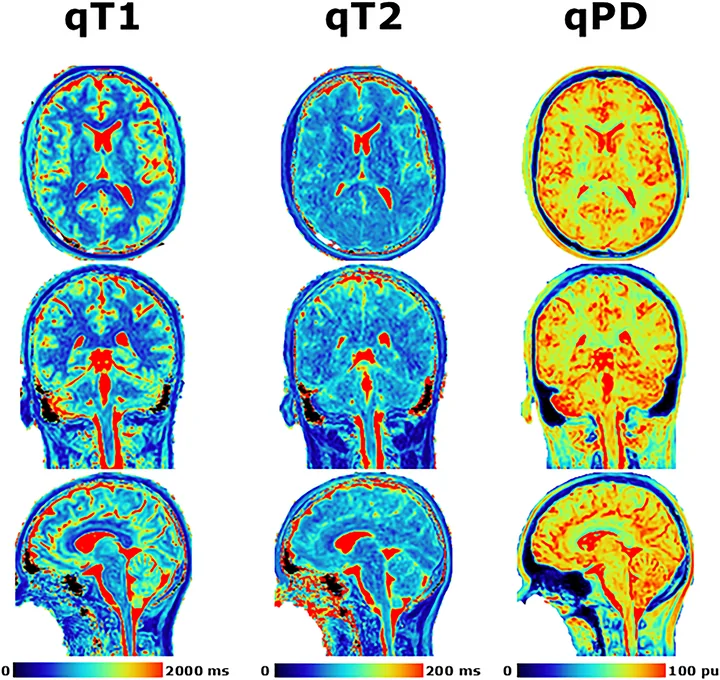
Demonstration of post‐processing available from SyMRI combined with 3D‐QALAS at high isotropic resolution in a healthy volunteer. Shown are the quantitative T1 (left), quantitative T2 (middle), and quantitative PD (right) in the axial (top), coronal (middle), and sagittal (bottom) orientations.

The quantitative results from Figure 5 for the automatically delineated white matter, grey matter, and whole brain are shown in Table 2. Compared to reference values from Fujita et al.,^21^ which used the 3D‐QALAS technique on a 1.5T Philips Ingenia MRI, the relative volume of the white matter was slightly lower for the MR‐Linac 3D‐QALAS sequence. The quantitative T1 values from the MR‐Linac 3D‐QALAS sequence were slightly higher than the reference values while the quantitative T2 and PD values were nearly identical. All CoV of the quantitative metrics were below 5% except for the volume measurements which is expected due to size differences in the volunteers.

**TABLE 2:** The calculated total relative structure volume compared to the whole brain and mean (CoV) of the quantitative T1, T2, and PD values inside the white matter and grey matter across all five volunteers. Also shown are the respective reference values.

	White Matter		Grey Matter	
	Value	CoV	Value	CoV
Relative Volume				
Measured	36%	8%	52%	7%
Reference	41%	5%	48%	5%
T1				
Measured	713 ms	2%	1166 ms	4%
Reference	580 ms	3%	910 ms	2%
T2				
Measured	76 ms	3%	88 ms	3%
Reference	78 ms	1%	88 ms	1%
PD				
Measured	73 pu	1%	83 pu	0.3%
Reference	65 pu	0.6%	77 pu	0.9%

The synthetic T1‐weighted, T2‐weighted, T2‐weighted fluid attenuated inversion recovery (FLAIR), proton density‐weighted, phase‐sensitive inversion recovery (PSIR), and double inversion recovery (DIR) with white matter suppression images in axial, coronal, and sagittal orientations are shown in Figure 6. The T1‐weighted images appeared to have the lowest amount of noise with the most noise seen in the T2‐weighted images. Adding the FLAIR component led to good CSF suppression with a very small amount of residual high intensity regions where CSF is expected. Only a moderate amount of noise was seen in the PD‐weighted images while effectively providing an average of the T1‐weighted and T2‐weighted images. Finally, moderate noise was seen in the PSIR images while providing a unique contrast for structure delineation while minimal noise was seen in the DIR images with mostly effective white matter suppression. An expansion of the synthetic T1‐weighted image across three axial, sagittal, and coronal slices is shown in Figure S1.

**FIGURE 6:**
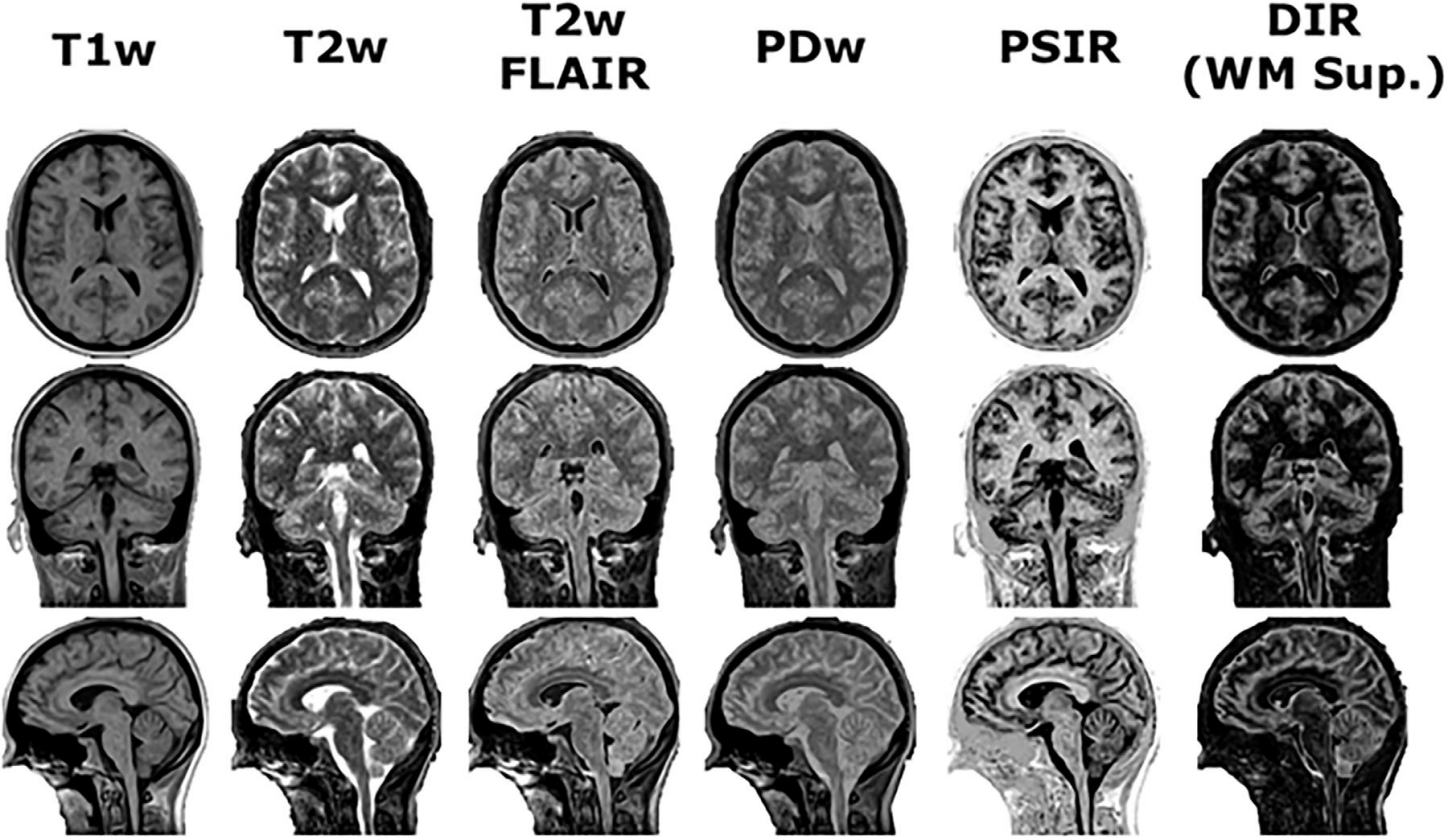
Demonstration of post‐processing available from SyMRI combined with 3D‐QALAS at high isotropic resolution in a healthy volunteer. Shown from left to right are the T1‐weighted, T2‐weighted, T2‐weighted fluid attenuated inversion recovery (FLAIR), proton density‐weighted, phase‐sensitive inversion recovery (PSIR), and double inversion recovery (DIR) with white matter suppression synthetic images in the axial (top), coronal (middle), and sagittal (bottom) orientations.

The calculated white matter, grey matter, cerebrospinal fluid, myelin, and non‐aqueous component synthetic images in axial, coronal, and sagittal orientations overlayed onto the synthetic T1‐weighted image is shown in Figure 7. Both white matter and grey matter appear in the correct locations of the brain, however some decreased confidence (i.e., a considerable number of voxels are below 100%) is seen particularly in the superior port of the images. The CSF map provides a clear distinction around both the sulci and gyri as well as the ventricle with high confidence. Similar to the white and grey matter, the myelin images have decreased confidence throughout the image due to the noise in the quantitative T2 maps. This noise is also generally reflected in the non‐aqueous content image as well.

**FIGURE 7:**
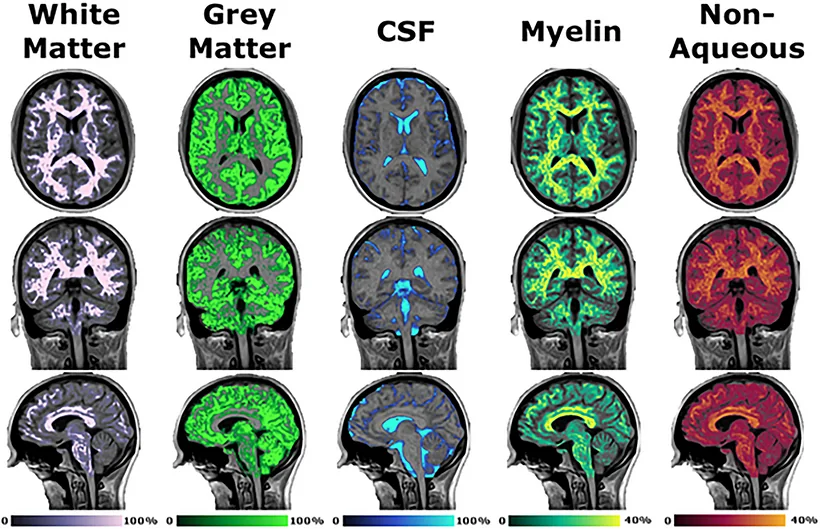
Demonstration of post‐processing available from SyMRI combined with 3D‐QALAS at high isotropic resolution in a healthy volunteer. Shown from left to right are the white matter, grey matter, cerebrospinal fluid, myelin, and non‐aqueous component synthetic images in the axial (top), coronal (middle), and sagittal (bottom) orientations overlayed onto the synthetic T1‐weighted image.

A visualization of the measured MyCPF, BPF, CSF, MyC, BPV, and ICV values in an example healthy volunteer in their 20s compared to age‐dependent reference values are shown in Figure 8. The measured value across all six metrics was within the reference range for healthy individuals in their 20s. Quantitatively, all metrics across each of the volunteers stayed within reference value ranges except for two individuals for MyC and one for ICV.

**FIGURE 8:**
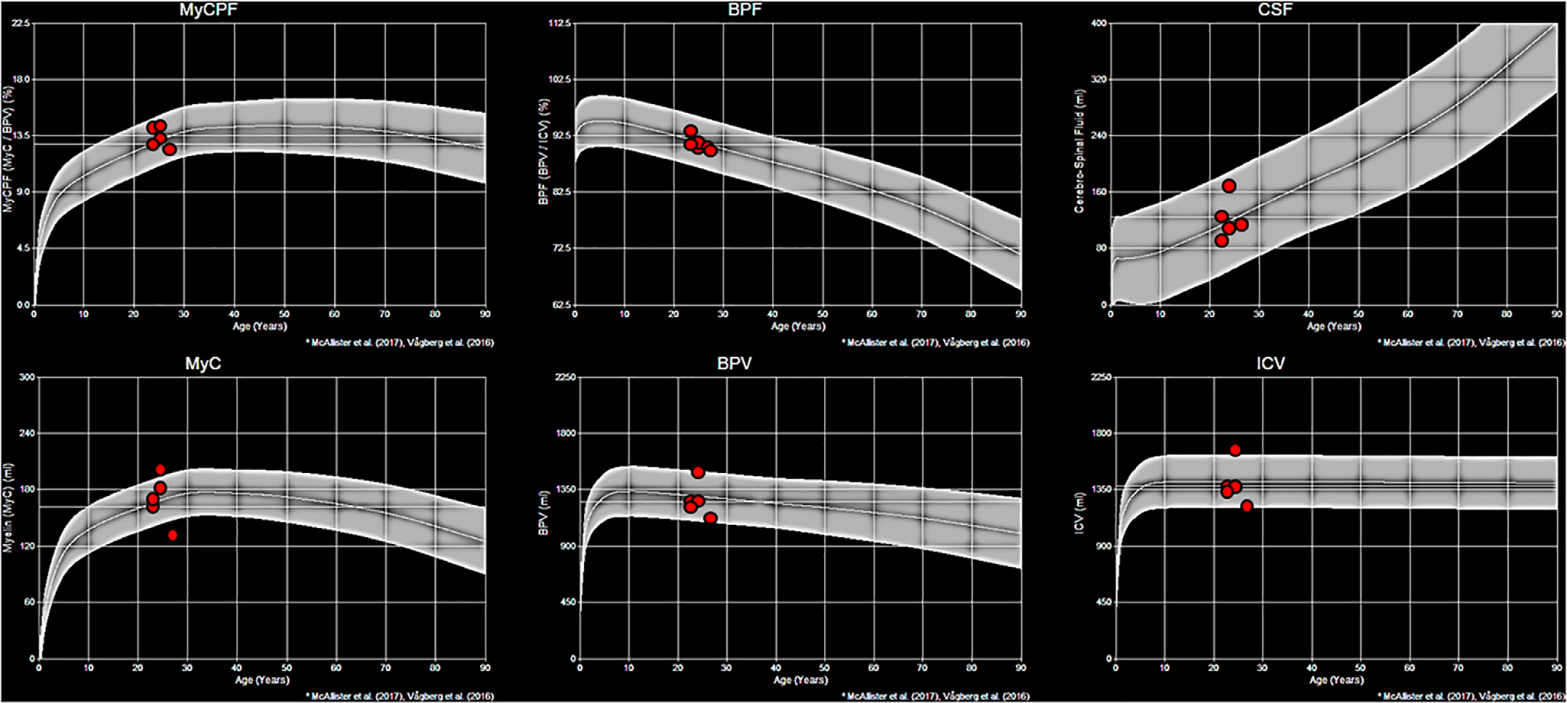
Demonstration of post‐processing available from SyMRI combined with 3D‐QALAS at high isotropic resolution for each healthy volunteer. Shown are the MyCPF, BPF, CSF, MyC, BPV, and ICV in the healthy volunteers in their 20s. The red dot is the measured content against the healthy volunteer's age while the grey bands are the predicted reference values for healthy individuals.

## 4 DISCUSSION

### 4.1 Summary

This study demonstrated the technical feasibility of the 3D‐QALAS acquisition on the 1.5T MR‐Linac by assessing its geometric accuracy, concordance, repeatability, and reproducibility in both phantoms and five healthy volunteers. The slope and LCCC between the measured and phantom reference values was 1.02 (LCCC = 1.00), 1.09 (LCCC = 0.90), and 0.99 (LCCC = 1.00) for the quantitative T1, T2, and PD values, respectively. The 3D‐QALAS quantitative T1 values of less than 1500 ms exhibited a consistent positive 50 ms systematic bias while higher values were slightly underestimated. Similarly, the quantitative T2 values showed general agreement up to 100 ms where they overestimated phantom reference values by nearly 20%. The median distortion remained below 2 mm, or under two voxels at distances of up to 90 mm from the center of the field‐of‐view. The repeatability and reproducibility CoV was under 5% and 8%, respectively, for all measured values in phantoms.

The white matter measured volumes from the MR‐Linac 3D‐QALAS were lower than the reference volumes, most likely due to the increased noise which led to holes in the segmentation, thus decreasing its final volume, though the CoV is large for each. The simultaneous decrease in white matter volume and increase in grey matter volume from the MR‐Linac 3D‐QALAS sequence could be due to overestimation of white matter quantitative T1 leading it to be misclassified as grey matter. Regardless, the measured brain volumes in the healthy volunteers were within expected age‐adjusted reference values.

### 4.2 Limitations and future work

The method to determine geometric distortion had limitations of precision due to 3D Slicer only allowing segmentations to be drawn on finite voxels, not partial voxels. Therefore, after a 0 mm distortion, the next lowest distortion would be 1 mm due to the 1 mm reconstructed voxel resolution of our 3D‐QALAS acquisition. Therefore, a median geometric distortion of between 1 – 2 mm across the entire fiducial array should be acceptable for most applications since it is only at the order of 1 – 2 voxels. Future work should be done to acquire 3D‐QALAS at higher spatial resolutions to improve this precision or use a software that allows partial voxel segmentations. It should be noted that although our reconstructed spatial resolution was 1 mm, our acquired resolution was 2 mm which means smaller features may be blurred due to partial volume effects. This may have consequences for more strict SRS cases where an accuracy of less than 1 mm is desired. Direct reproducibility across multiple 1.5T MR‐Linac machines could not be determined since our institution only has one, therefore further work should be done to confirm the results shown here in addition to test‐retest volunteer imaging on the same system.

Although efficient compared to traditional quantitative relaxometry mapping techniques, a total acquisition time of over seven minutes is higher than desired and should be reduced in further studies. A current hinderance to further reducing the acquisition time is the compressed sensing (CS‐SENSE) factor which would lead to the introduction of image noise.^44^ However, combining 3D‐QALAS with Wave–Controlled Aliasing In Parallel Imaging (Wave‐CAIPI^45^) readouts can reduce and unify g‐factors,^46^ especially when combined with model‐based deep learning (Wave‐MoDL^47^) and zero‐shot deep learning subspaces (Zero‐DeepSub^48^). Another approach is to use offline compressed sensing reconstruction using the raw image data which has been previously demonstrated successfully on the 1.5T MR‐Linac for quantitative T1 mapping using non‐Cartesian acquisitions.^49^ This would help reduce the amount of data required during acquisition, thus reducing acquisition time, with the tradeoff of longer reconstruction times compared to using the scanner's built‐in reconstruction.

The quantitative T2 values from the 3D‐QALAS sequence exhibited nearly 20% positive bias compared to phantom reference values at T2 values above 100 ms. This may be a concern for accurately identifying grey matter which has T2 values around 100 ms in healthy individuals.^50^ In this study, the grey matter maps were generally anatomically accurate given the acquired spatial resolution of 2 mm, however, there were some holes seen in the segmentation due to image noise. Due to these concerns, future work should focus on optimization of the acquisition parameters^51^ to better quantify relaxometric properties for tissues in the brain, preferably in an R‐IDEAL Stage 2a study. Some acquisition parameters that should be focused on during the optimization would be the TFE inversion prepulse delay time which would shift the location of the first readout following the inversion pulse primarily affecting the T1 quantification, the T2‐preparation delay time which would affect the T2 quantification, and the internal trigger delay time which would adjust the spacing between readouts primarily affecting the T1 quantification. These optimizations could also minimize the inhomogeneities seen in the quantitative T2 maps in Figure 1. Further optimization could then be made to the CS‐SENSE factor and its denoising factor for reduced acquisition times, larger field‐of‐views, and increased signal‐to‐noise ratio (SNR)—especially since the water‐fat shift (WFS) must be minimum to minimize chemical shift induced signal intensity biases.^52^ From a clinical perspective, reducing the variations in the quantitative T1, T2, and PD values would lead to more confident decision making for adaptive therapies since the quantitative difference between two measurements needed to see a “true change” would be reduced. Secondly, improved quantification of the relaxation parameters would lead to more accurate synthetic contrast maps and subsequent segmentations.

### 4.3 Extended applications

Building on the work presented here investigating 3D‐QALAS, this high resolution isotropic method to quantitatively map multiparametric relaxometric properties can be applied synergistically with recent efforts to define probabilistic tumor spread.^53^ This approach, in conjunction with the high temporal resolution of the MR‐Linac, can bring new insights into how tumors and OOIs respond to radiation therapy allowing for advanced tumor control probability (TCP^54^) and normal tissue complication probability (NTCP^55^) modeling. Currently, the 3D‐QALAS sequence utilizes a one echo readout, however multiple echoes can be added which would allow for quantitative T2* mapping and it's relative derived maps such as in the Multiple‐echoes with Optimized Simultaneous Acquisition (MIMOSA) technique.^56^ Another approach is to add an additional T2‐preparation module which can be employed to more accurately quantify the myelin water fraction (MWF) as demonstrated in the MWF‐QALAS^57^ and Omni‐QALAS^58^ techniques. Another interesting future application of 3D‐QALAS on the MR‐Linac is the reconstruction of time‐of‐flight MR angiography images using deep learning^59^ which can be used for improved target planning.^60^, ^61^, ^62^ Finally, the generation of synthetic computed tomography (CT) images^63^ at 1 mm isotropic reconstructed resolution derived from the three quantitative maps from 3D‐QALAS would help MR‐only workflows,^64^, ^65^, ^66^ reducing burden on patients while minimizing delay in care. Integration of these advanced, high resolution, quantitative MRI techniques into the adaptive radiation therapy workflow of the brain has the potential to help guide the reduction of target margins,^67^ especially when combined with prospective motion correction techniques.^68^

## 5 CONCLUSION

The 3D‐QALAS sequence is a promising approach to acquire multiparametric quantitative MRI relaxometric maps and synthetically generated contrast‐weighted maps at high resolution isotropic voxel sizes. In this paper, we demonstrated the potential of 3D‐QALAS to transform the quantitative image biomarker capacity of the 1.5T MR‐Linac in the following ways: 1) achievement of quantitative T1 / T2 / PD maps at 1 mm isotropic voxel size in under eight minutes, 2) derivation of pre‐registered synthetic contrast‐weighted images at equivalent spatial resolution, and 3) repeatability under 5%, reproducibility under 8%, and median spatial accuracy under 2 mm. With this technique, a new era is emerging where quantitative MRI maps can be acquired at the same spatial resolution as traditional anatomical imaging (i.e., T2‐weighted), enabling advanced tumor and OOI quantification and characterization, ideal for adaptive radiation therapy applications on the MR‐Linac.

## AUTHOR CONTRIBUTIONS

Contributor Roles Taxonomy (CRediT) Attribution Statement: Conceptualization: Lucas McCullum and Clifton D. Fuller; Data curation: Lucas McCullum, Ashley Harrington, Natalie West, Grace Nehring, Ziyu Fu, Ergys Subashi, and Ken‐Pin Hwang; Formal analysis: Lucas McCullum and Ashley Harrington; Funding acquisition: Clifton D. Fuller; Investigation: Lucas McCullum and Ashley Harrington; Methodology: Lucas McCullum and Clifton D. Fuller; Project administration: Clifton D. Fuller; Resources: Lucas McCullum, Ashley Harrington, Brian A. Taylor, Ken‐Pin Hwang and Clifton D. Fuller; Software: Lucas McCullum; Supervision: Brian A. Taylor, Ken‐Pin Hwang and Clifton D. Fuller; Validation: Lucas McCullum; Visualization: Lucas McCullum; Writing—original draft: Lucas McCullum; Writing—review & editing: Lucas McCullum, Ashley Harrington, Natalie West, Grace Nehring, Ziyu Fu, Ergys Subashi, Brian A. Taylor, Ken‐Pin Hwang and Clifton D. Fuller.

## FUNDING INFORMATION

Lucas McCullum is supported by a National Institutes of Health (NIH) Diversity Supplement (R01CA257814‐02S2) and the Larry Deaven Ph.D. Fellowship in Biomedical Sciences. BAT is supported by a National Institutes of Health Career Development Award (K23DA049216). Clifton D. Fuller received funding and salary support from the National Science Foundation (NSF)/National Institutes of Health (NIH) National Cancer Institute (NCI) via the Smart and Connected Health (SCH) Program (R01CA257814). Clifton D. Fuller has also received funding and program support from the NIH National Institute of Dental and Craniofacial Research (NIDCR) Academic‐Industrial Partnership (R01DE028290), and the NIH NCI MD Anderson Cancer Center Support Grant (CCSG) Program (P30CA016672).

## CONFLICT OF INTEREST STATEMENT

Lucas McCullum has received unrelated travel / hotel accommodations from Elekta AB. Ken‐Pin Hwang has received related investigational software / research support from SyntheticMR AB and unrelated research support from GE Healthcare. Clifton D. Fuller has received unrelated grant support from Elekta AB and holds unrelated patents licensed to Kallisio, Inc. (US PTO 11730561) through the University of Texas, from which they receive patent royalties. Clifton D. Fuller has received unrelated grant support from Elekta AB and holds unrelated patents licensed to Kallisio, Inc. (US PTO 11730561) through the University of Texas, from which they receive patent royalties. Clifton D. Fuller has also received unrelated travel and honoraria from Elekta AB, Philips Medical Systems, Siemens Healthineers/Varian, and GE Healthcare. Clifton D. Fuller receives unrelated salary support as an NIH sub‐awardee through NRG Oncology.

## ETHICS STATEMENT

All participants provided written informed consent. Volunteers were consented to an internal volunteer imaging protocol (PA15‐0418), both approved by the institutional review board at The University of Texas MD Anderson Cancer Center.

## Supporting information




Supporting Information

Supporting Information

## Data Availability

All relevant image files and contours in NIfTI format are to be made publicly available after manuscript acceptance at the following FigShare URL: https://doi.org/10.6084/m9.figshare.31594819. The accompanying code for image visualization and statistical analysis will be made publicly available at the following URL: https://github.com/Lucas‐Mc/QALAS‐MRL_R‐IDEAL_0‐1.
