# Supplementary material for: Technical development and implementation of 3D‐QALAS on a 1.5T MR‐Linac for the brain: A prospective R‐IDEAL stage 0/1 technology development report
Source: J Appl Clin Med Phys. 2026 Oct 28;27(11):e70886. doi: 10.1002/acm2.70886 (PMC13715509; doi:10.1002/acm2.70886)
Supplement: Supplementary file 1 [file ACM2-27-e70886-s002.docx]

**Supplementary Materials**

**Fig. S1.** An example synthetic T1-weighted image generated from SyMRI from a healthy volunteer in this study showing three axial slices (top row), three sagittal slices (middle row), and three coronal slices (bottom row).
